# Mast cell hyperactivity underpins the development of oxygen-induced retinopathy

**DOI:** 10.1172/JCI89893

**Published:** 2017-10-09

**Authors:** Kenshiro Matsuda, Noriko Okamoto, Masatoshi Kondo, Peter D. Arkwright, Kaoru Karasawa, Saori Ishizaka, Shinichi Yokota, Akira Matsuda, Kyungsook Jung, Kumiko Oida, Yosuke Amagai, Hyosun Jang, Eiichiro Noda, Ryota Kakinuma, Koujirou Yasui, Uiko Kaku, Yasuo Mori, Nobuyuki Onai, Toshiaki Ohteki, Akane Tanaka, Hiroshi Matsuda

**Affiliations:** 1 Cooperative Major in Advanced Health Science, Graduate School of Bio-Applications and System Engineering, Tokyo University of Agriculture and Technology, Tokyo, Japan.; 2 Laboratory of Veterinary Molecular Pathology and Therapeutics, and Division of Animal Life Science, Institute of Agriculture, Tokyo University of Agriculture and Technology, Tokyo, Japan.; 3 Department of Neonatology and Tokyo Metropolitan Children’s Medical Center, Tokyo, Japan.; 4 Institute of Inflammation and Repair, University of Manchester, Royal Manchester Children’s Hospital, Manchester, United Kingdom.; 5 Tokyo Biomarker Innovation Research Association, Tokyo, Japan.; 6 Department of Ophthalmology, Tokyo Metropolitan Children’s Medical Center, Tokyo, Japan.; 7 Laboratory of Comparative Animal Medicine, Division of Animal Life Science, Institute of Agriculture, Tokyo University of Agriculture and Technology, Tokyo, Japan.; 8 Department of Synthetic Chemistry and Biological Chemistry, Graduate School of Engineering, Kyoto University, Kyoto, Japan.; 9 Department of Biodefense Research, Medical Research Institute, Tokyo Medical and Dental University, Tokyo, Japan.

**Keywords:** Angiogenesis, Inflammation, Mast cells, Mouse models

## Abstract

Mast cells are classically thought to play an important role in protection against helminth infections and in the induction of allergic diseases; however, recent studies indicate that these cells also contribute to neovascularization, which is critical for tissue remodeling, chronic inflammation, and carcinogenesis. Here, we demonstrate that mast cells are essential for sprouting angiogenesis in a murine model of oxygen-induced retinopathy (OIR). Although mouse strains lacking mast cells did not exhibit retinal neovascularization following hypoxia, these mice developed OIR following infusion of mast cells or after injection of mast cell tryptase (MCT). Relative hypoxia stimulated mast cell degranulation via transient receptor potential ankyrin 1. Subsequent surges in MCT stimulated retinal endothelial cells to produce monocyte chemotactic protein-1 (MCP1) and angiogenic factors, leading to sprouting angiogenesis. Mast cell stabilizers as well as specific tryptase and MCP1 inhibitors prevented the development of OIR in WT mice. Preterm infants with early retinopathy of prematurity had markedly higher plasma MCT levels than age-matched infants without disease, suggesting mast cells contribute to human disease. Together, these results suggest therapies that suppress mast cell activity should be further explored as a potential option for preventing eye diseases and subsequent blindness induced by neovascularization.

## Introduction

Mast cells are important for innate immune defense against microbes and parasites (1, 2). A number of toxins and chemicals can induce host-protective or pathogenic activation of mast cells (3, 4). Paul Ehrlich, in his 1878 doctoral thesis, was the first to describe mast cells (Mastzellen), noting that they often congregate around blood vessels of tissues (5). Mast cells accumulate around expanding neoplasms and correlate with the degree of tumor vascularization (6, 7). After activation, mast cells release a number of proinflammatory cytokines and angiogenic mediators as well as proteolytic enzymes, including tryptase and chymase (8, 9). Because of their multipotency, mast cell–derived factors can induce both tissue damage and remodeling.

The eye provides a window into the process of vascularization in both health and disease. Ocular neovascularization is the leading cause of blindness in adults, for instance, in age-related macular degeneration and diabetic retinopathy (10). In infants and children, retinopathy of prematurity (ROP) is also a leading cause of blindness, induced by oxygen-dependent retinal vascular injury and occlusion, and in severe cases (stage 3+), subsequent neovascularization, with a risk of retinal scarring and detachment (11–13). ROP only develops in preterm infants, particularly those at the extremes of survival (45% of infants born at 22–23 weeks gestation compared with 0.1% of infants born at 30–31 weeks gestation) and thus provides an insight at the interface between normal and abnormal vascular development. Excessive oxygen therapy given to extremely preterm infants is the key risk factor for ROP (14–16). It results in vascular occlusion and subsequent reactive neovascularization mediated by VEGF, which can be partly offset using anti-VEGF monoclonal therapy (bevacizumab) (17). However, some ROP patients still end up with visual impairment, even after treatment (18). The precise mechanism by which aberrations in infants’ oxygen tension triggers neovascularization is currently unclear.

Tissue vascularization begins with vasculogenesis, the creation of new vessels from angioblasts (19). Subsequent weaknesses in the supporting vascular basement membrane and extracellular matrix at intervals along these vessels result in secondary branches or sprouting angiogenesis (20, 21). Abnormal vascularization is recognized to be an integral part of chronic inflammatory response, tissue remodeling, and carcinogenesis (22, 23). Serine proteases, VEGF, and basic FGF, which are released by mast cells, have been reported to facilitate neovascular sprouts and promote normal and tumor-associated angiogenesis (7). In the current study, we investigated the role of mast cells in retinal neovascularization during alterations in oxygen tension using well-established mouse models of oxygen-induced retinopathy (OIR).

## Results

### Mast cells are essential for retinal neovascularization in OIR.

**Figure 1:**
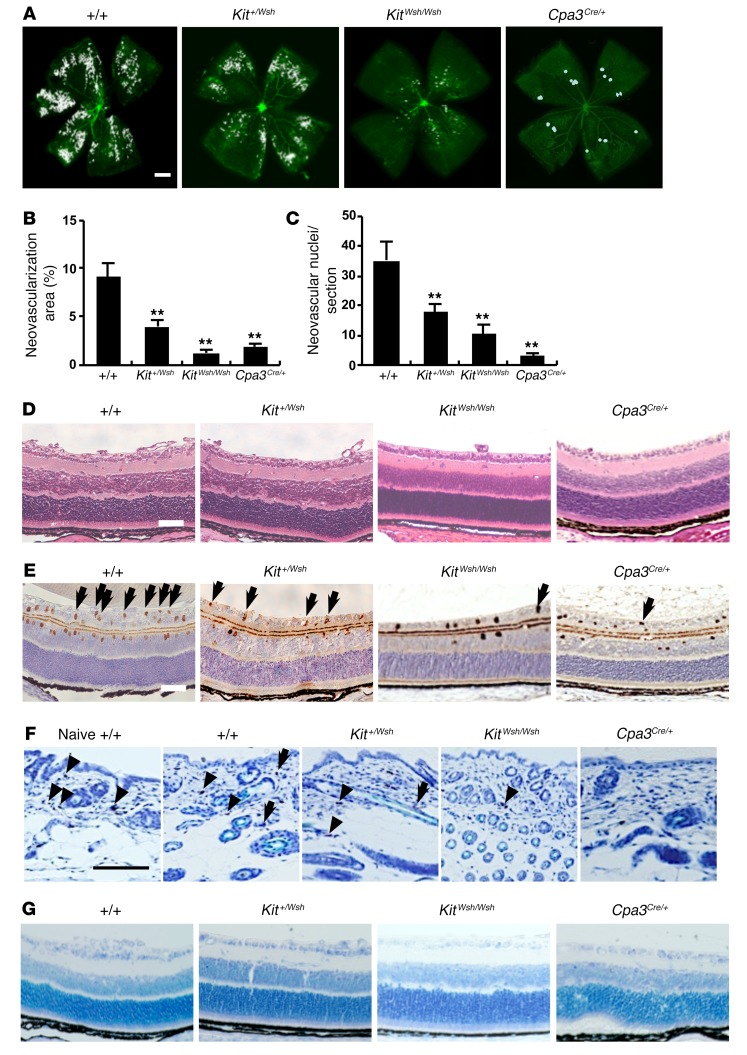
Mast cell deficiency prevented in the development of retinal neovascularization in an OIR mouse model. (**A** and **B**) Whole-mounted retinas revealed that pathological neovascularization, shown as tufts (white areas), was induced in mast cell–sufficient WT mice, but not in mast cell–deficient mice on P17. *n* = 8 in each group. **P < 0.01 versus WT mice, Dunnett’s test. (**C**) Retinal neovascularization on P17 was quantified by counting the number of neovascular cell nuclei at the retinal inner surface of eye sections after H&E staining. The number of neovascular nuclei was lower in *Kit^+/Wsh^*, *Kit^Wsh/Wsh^*, and *Cpa3^Cre/+^* mice than in WT mice. *n* = 8 in each group. **P < 0.01 versus WT mice, Dunnett’s test. (**D**–**G**) Cross-sectional analysis of retinas was performed by H&E (**D**), PECAM-1 (**E**), or toluidine blue (**F**) staining of formalin-fixed paraffin-embedded sections. Results are representative of 3 independent experiments. (**E**) Arrows indicate endothelial cells that have penetrated into the vitreous space. Toluidine blue staining showed mast cells in the dorsal skin (**F**) of WT and *Kit^+/Wsh^* mice, but not in the retina (**G**). Arrows and arrowheads indicate degranulated and nondegranulated mast cells, respectively (**F**). Scale bars: 500 μm (**A**); 100 μm (**D**–**G**). Results are shown as mean ± SEM of values determined from 3 independent experiments (**B** and **C**).

We initially used a murine model of OIR (24) to examine the role of mast cells in the development of retinal neovascularization in *Kit^Wsh/Wsh^* and *Cpa3^Cre/+^* mast cell–deficient mice (25, 26) (Figure 1). *Kit^Wsh/Wsh^* mice carry a mutation in *Kit* that results in mast cell deficiency. Feyerabend et al. (26) established the *Cpa3^Cre/+^* mast cell–deficient mice by depleting 28 nucleotides in the first exon of the mast cell carboxypeptidase A3 locus (*Cpa3*) and by targeted insertion of Cre-recombinase. Heterozygous *Cpa3^Cre/+^* mice totally lacked mast cells in connective and mucosal tissues via a genotoxic Trp53-dependent mechanism. Whole-mount analysis showed that hyperoxic exposure for 5 days from P7 to P12 resulted in vascular occlusion in the central part of the retina in all mice on P12. In WT mice, after a further 5 days, neovascular sprouts and tufts developed, a hallmark of ROP in humans (27) (Figure 1, A–D). These neovascular tufts and nuclei were markedly decreased in *Kit^Wsh/Wsh^* and *Cpa3^Cre/+^* mice (Figure 1, A–D), while an intermediate number of neovascular nuclei was found in *Kit^+/Wsh^* mice (Figure 1, A–D). Penetration of endothelial cells positive for PECAM-1 into the vitreous was also very low in mast cell–deficient mice (Figure 1E). No neovascularization was observed in any of the mice exposed to only room air (data not shown). In WT mice and *Kit^+/Wsh^* mice, mast cells were observed in the dorsal skin on P17 and 40% of the skin mast cells had degranulated (Figure 1F and Table 1). In contrast, no or very few mast cells could be detected in the skin of mast cell–deficient mice (Figure 1F and Table 1). No mast cells were observed in the retina of all the mice (Figure 1G).

**Figure 2:**
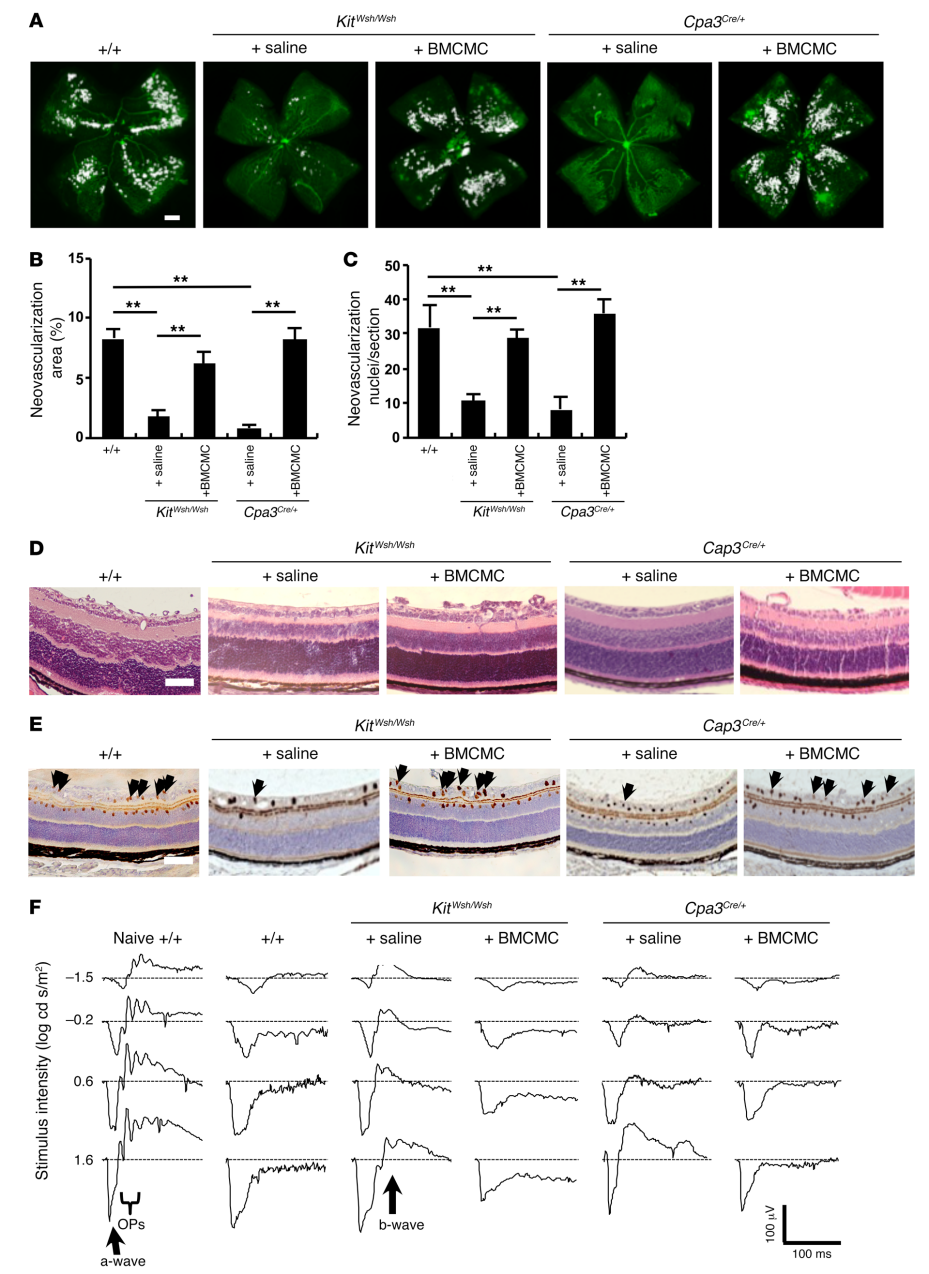
Injection of mast cells induced the formation of neovascular tufts in mast cell–deficient mice. (**A** and **B**) BMCMC but not saline treatment induced the formation of new abnormal blood vessels (white areas) in mast cell–deficient mice with OIR on P17. *n* = 8 in each group. **P < 0.01 versus saline-injected mast cell–deficient mice, 1-way ANOVA with Tukey’s test. (**C**) Retinal neovascularization was quantified on P17 by counting the number of neovascular nuclei extending into the vitreous after H&E staining. The number of neovascular nuclei in BMCMC-injected mast cell–deficient pups was comparable to that in WT mice. *n* = 8 in each group. **P < 0.01 versus saline-injected mast cell–deficient mice, 1-way ANOVA with Tukey’s test. (**D** and **E**) Cross-sectional analysis of retinas was performed by H&E (**D**) or PECAM-1 (**E**) staining of formalin-fixed paraffin-embedded sections. Arrows indicate endothelial cells that have penetrated into the vitreous space. Results are representative of 3 independent experiments. (**F**) Normal ERG responses on P19 were seen in age-matched naive WT and saline-injected mast cell–deficient mice, but not in WT or BMCMC-injected mast cell–deficient mice. Scale bars: 500 μm (**A**); 100 μm (**D** and **E**). Results are shown as mean ± SEM of values determined from 3 to 4 independent experiments (**B** and **C**).

As more direct evidence that mast cells are involved in the pathogenesis of OIR, BM-derived cultured mast cells (BMCMCs) (28) were injected into the peritoneal cavity of *Kit^Wsh/Wsh^* and *Cpa3^Cre/+^* mice on P1 or P2. I.p. injection of BMCMCs into mast cell–deficient mice resulted in neovascular tufts similar in extent to those observed in WT mice on P17 (Figure 2, A and B). H&E staining demonstrated that the numbers of neovascular nuclei were increased in *Kit^Wsh/Wsh^* and *Cpa3^Cre/+^* mice injected with BMCMCs compared with those of mice injected with saline alone (Figure 2, C and D). In addition, PECAM-1–positive endothelial cells were found to extend into the vitreous after the injection of BMCMCs into mast cell–deficient mice (Figure 2E).

**Figure 3:**
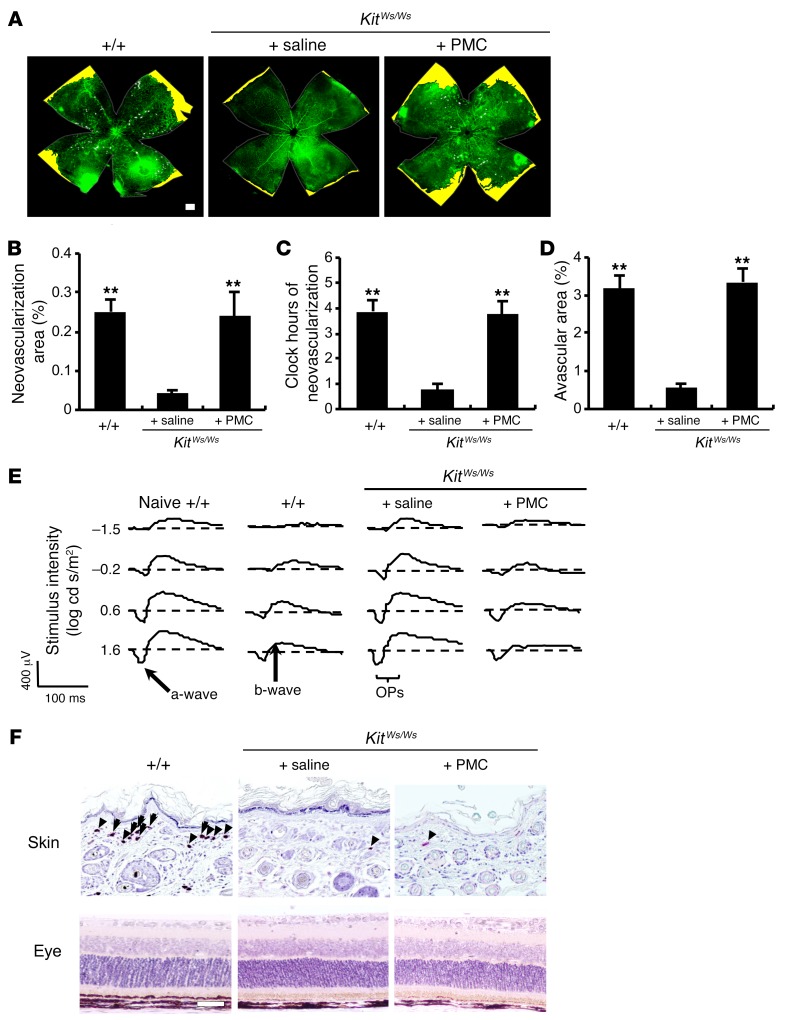
Injection of mast cells restored retinal neovascularization in mast cell–deficient DA/Ham-*Kit^Ws/Ws^* rats. (**A**) Whole-mount analysis of the rat ROP model was conducted on P17. Neovascular tufts are shown as white areas. Yellow areas indicate avascular areas. (**B**–**D**) Quantification of whole-mount analysis showed that neovascularization was rescued in *Kit^Ws/Ws^* pups injected with 1 × 10^6^ peritoneal mast cells (PMC) freshly isolated from WT rats. *n* = 8 in each group. **P < 0.01 versus saline-injected *Kit^Ws/Ws^* rats, Dunnett’s test. (**E**) ERG analysis was performed on P19. (**F**) Chloroacetate esterase–positive mast cells were observed in the dorsal skin, but not in the retina. Arrows and arrowheads indicate degranulated and nondegranulated mast cells, respectively. Results are representative of 3 independent experiments. Scale bars: 500 μm (**A**); 100 μm (**F**). Results are shown as mean ± SEM of values determined from 4 independent experiments (**B**–**D**).

Retinal function is markedly impaired in mice with OIR, as measured by decreased b-wave amplitude (29). To assess the retinal function in our murine OIR model, we analyzed single-flash electroretinogram (ERG) patterns on P19. ERG analyses revealed that, while WT and BMCMC-injected mast cell–deficient mice had complete loss of b-waves and oscillatory potential–waves (OP-waves), mast cell–deficient mice injected with saline had normal b- and OP-waves, comparable to those of naive WT mice (Figure 2F and Table 2). Because there are some differences between the mouse and rat OIR models (30), we also performed experiments using mast cell–deficient *Kit^Ws/Ws^* rats (31). The results of the rat model were in keeping with those of the murine model (Figure 3 and Table 2).

### Transient receptor potential ankyrin 1 is responsible for mast cell degranulation induced by relative hypoxia.

**Figure 4:**
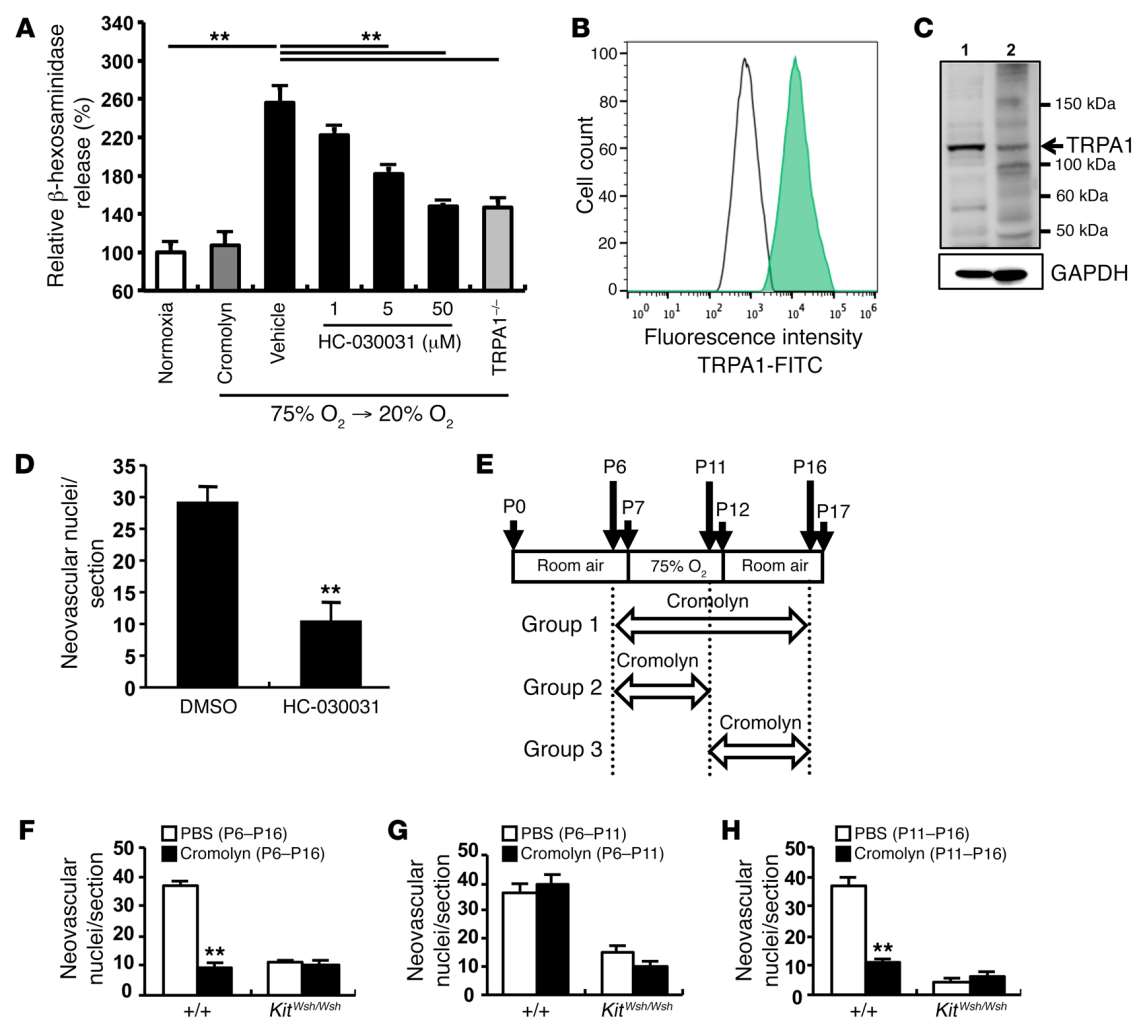
Oxygen-induced degranulation of mast cells was induced by TRPA1. (**A**) When BMCMCs were moved from 75% oxygen to 20% oxygen, significant degranulation was induced. TRPA1 inhibitor HC-030031 or TRPA1 deficiency suppressed relative hypoxia-induced degranulation of BMCMCs. **P < 0.01 versus vehicle, Dunnett’s test. Typical results with 5 measurements of 3 independent experiments are shown as mean ± SEM. (**B** and **C**) To identify O_2_-sensing molecules on mast cells, TRPA1 was detected by flow cytometry and immunoblotting. Results are representative of 3 independent experiments. A green peak shows a TRPA1-positive reaction and an open peak shows reactivity of a control (**B**). Instead of first Abs, rabbit serum was used as a control. Strong positive reactions that were visualized around 110 kDa (indicated by an arrow) were estimated as TRPA1 proteins (**C**). Lane 1, mouse brain membrane; lane 2, C57BL/6 BMCMCs. GAPDH was used as a control for one of the cytosolic endogenous proteins. (**D**) TRPA1 inhibitor HC-030031 abrogated abnormal neovascularization in mice treated from P11 to P16. *n* = 8 in each group. **P < 0.01 versus DMSO-injected control mice, Mann-Whitney *U* test. (**E**–**H**) Mast cell stabilizer cromolyn suppressed retinal neovascularization in WT mice. (**E**) Schematic of the study design. WT and *Kit^Wsh/Wsh^* pups were given cromolyn or vehicle daily by i.p. injections. Mice were injected with cromolyn from P6 to P16, P6 to P11, or P11 to P16 in group 1, 2, or 3, respectively. Eyes were enucleated on P17. (**F**) Treatment with cromolyn from P6 to P16 reduced pathological neovascularization in WT mice. (**G**) Cromolyn did not affect abnormal neovascularization in mice in group 2. (**H**) Neovascular tufts were reduced in WT mice treated with cromolyn between P11 and P16, as in *Kit^Wsh/Wsh^* mice. *n* = 9 in each group. **P < 0.01 versus PBS-injected control mice, Mann-Whitney *U* test (**F**–**H**). Results are shown as mean ± SEM of values determined from 3 independent experiments (**D**, **F**–**H**).

To clarify the mechanism by which fluctuations in oxygen levels lead to mast cell degranulation, we measured β-hexosaminidase released from BMCMCs cultured in 75% oxygen for 5 days and then 20% oxygen for 12 hours in vitro. The protocol mimicked the in vivo experiments. Degranulation was induced after mast cells were moved from hyperoxic to normoxic conditions (Figure 4A), indicating that relative hypoxia triggered mast cell degranulation. As transient receptor potential ankyrin 1 (TRPA1) has been reported to act as an oxygen sensor in neural cells (32), we studied TRPA1 expression in mast cells. TRPA1 was expressed on BMCMCs, as demonstrated by flow cytometry (Figure 4B). TRPA1 from whole cell lysate of BMCMCs derived from C57BL/6 mice had a molecular weight of 110 kDa (Figure 4C). A TRPA1-specific inhibitor, HC-030031 (33), suppressed hypoxia-induced degranulation of mast cells in a dose-dependent manner (Figure 4A). To confirm the role of TRPA1 in relative hypoxia-induced degranulation of mast cells, we generated BMCMCs from BM cells isolated from TRPA1-deficient mice. Relative hypoxia-induced degranulation was markedly reduced in TRPA1-deficient BMCMCs (Figure 4A). These results indicate that TRPA1 mediates mast cell degranulation induced by relative hypoxia. To confirm the contribution of TRPA1 to oxygen-mediated mast cell degranulation in the OIR model, HC-030031 was administered daily to mice of the OIR model from P11 to P16, using the same protocol as use for group 3, shown in Figure 4E. Neovascular nuclei were markedly suppressed in HC-030031–treated OIR mice (Figure 4D), indicating that TRPA1 was essential for the development of OIR.

### The mast cell stabilizer cromolyn completely blocks retinal neovascularization induced by relative hypoxia.

Mast cell granule components have been reported to be potent stimulators of angiogenesis. Therefore, we next examined whether inhibition of mast cell degranulation reduced retinal neovascularization in vivo. Murine pups were injected daily with a mast cell stabilizer, cromolyn, under conditions of varying oxygen tension (Figure 4E). Administration of cromolyn for 10 days (P6–P16) completely inhibited the formation of neovascular tufts in WT mice (Figure 4F). As expected, suppression of mast cell degranulation by cromolyn for 5 days from the day before mice were moved to conditions of relative hypoxia (P11–P16) significantly decreased the number of endothelial nuclei that extended into the vitreous space (Figure 4H). In contrast, no suppressive effects of cromolyn were observed when it was just administered during the period of exposure to hyperoxia (P6–P11) (Figure 4G). Thus, the marked reduction in oxygen concentration is critical for mast cell activation and the development of OIR.

### Neovascularization associated with OIR is induced by mast cell tryptase.

**Figure 5:**
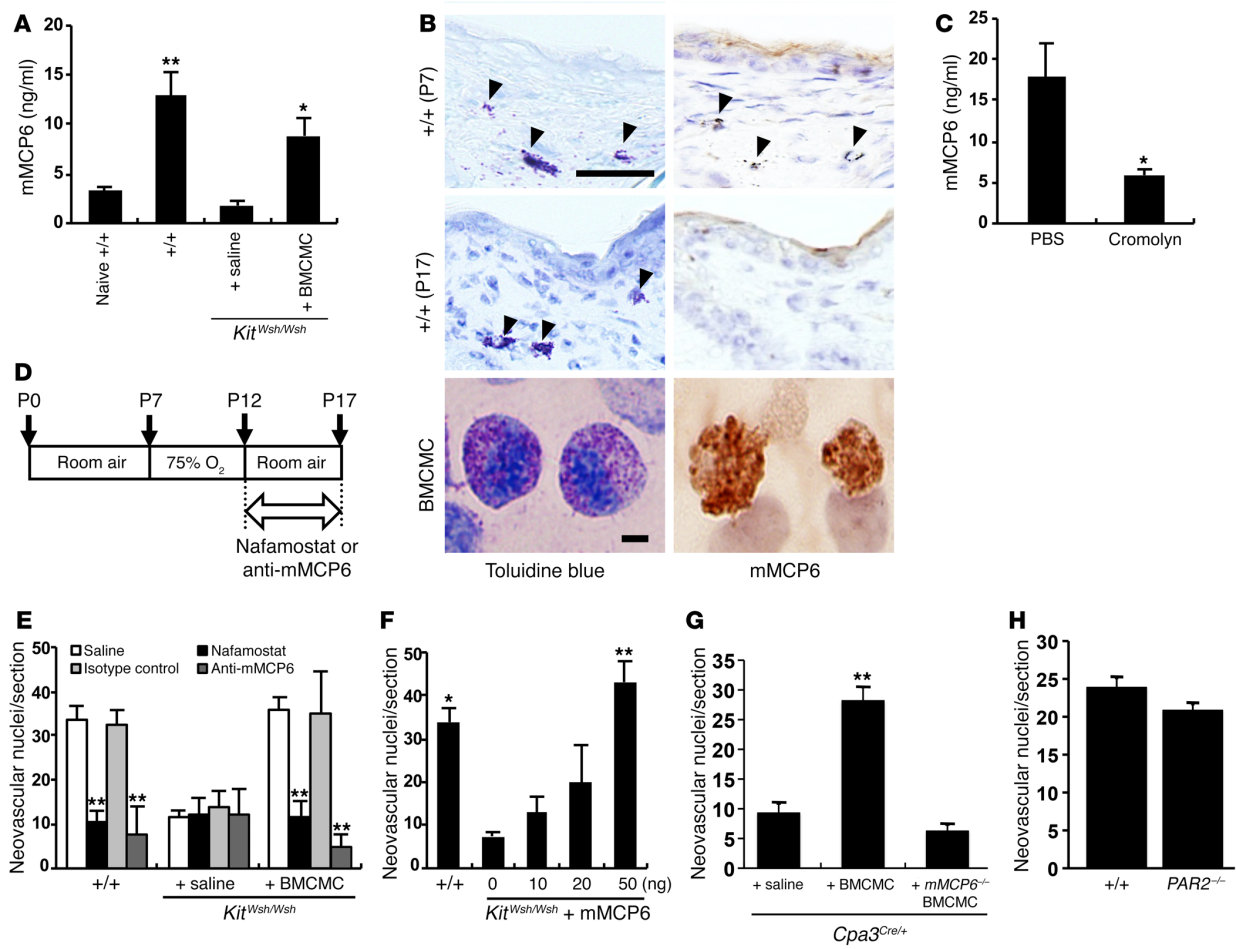
mMCP6 triggered retinal neovascularization. (**A**) mMCP6 ELISA was performed using serum from the OIR model and age-matched naive WT pups on P17. *n* = 8 in each group. *P < 0.05; **P < 0.01 versus saline-injected mice, Dunnett’s test. (**B**) mMCP6 immunohistochemical analysis was performed on skin sections of WT mice on P7 and P17 and on BMCMCs cultured for 5 weeks. Results are representative of 3 independent experiments. Arrowheads indicate mast cells in the skin. On P17, mast cells were degranulated and mMCP6 positivity was not identified. (**C**) mMCP6 ELISA was performed using mouse serum obtained at 6 hours after treatment with cromolyn on P12. *n* = 8 in each group. *P < 0.05 versus control mice, Mann-Whitney *U* test. (**D**) Schematic of the study design. Pups were i.p. injected with 20 μl NM or anti-mMCP6 mAbs every day from P12 to P17. Animals were sacrificed, and eyes were enucleated on P17. (**E**) Quantification of neovascular nuclei in mice treated with NM and anti-mMCP6 mAbs. *n* = 9 in each group. **P < 0.01 versus saline-injected mice, Dunnett’s test. (**F**) Quantification of neovascular nuclei in mice injected with recombinant mMCP6. *n* = 9 in each group. *P < 0.05; **P < 0.01 versus *Kit^Wsh/Wsh^* mice treated with vehicle alone, Dunnett’s test. (**G**) OIR was not induced by reconstitution of *Cpa3^Cre/+^* mice with mMCP6-deficient BMCMCs. *n* = 8 in each group. **P < 0.01 versus saline treatment, Dunnett’s test. (**H**) Retinal neovascularization in relative hypoxic condition in PAR2-deficient mice. *n* = 8 in each group. Scale bars: 50 μm (**B**, skins); 20 μm (**B**, BMCMC). Results are shown as mean ± SEM of values determined from 3 to 4 independent experiments (**A**, **C**, **E**–**H**).

Tryptase is the most abundant granule-derived serine protease in mast cells and has been reported to stimulate the proliferation of endothelial cells, promote tube formation, and degrade the connective tissue matrix to provide space for new vessel growth (8, 9). In mice, the major tryptases are mouse mast cell protease 6 (mMCP6) and mMCP7 (34). The C57BL/6 mouse strain lacks mMCP7 because of a spontaneous mutation within the *Mcp7* gene (35). We therefore investigated whether mMCP6 promoted retinal angiogenesis in the OIR mouse model. Raised serum mMCP6 levels were found in the OIR model of WT and BMCMC-injected *Kit^Wsh/Wsh^* pups compared with those of age-matched naive WT or saline-injected *Kit^Wsh/Wsh^* pups after the exposure to relative hypoxia (Figure 5A). Mast cells were identified in the dorsal skin of WT mice with OIR on P7, and degranulation of them was obvious on P17 (Figure 5B). mMCP6 was also detected in BMCMCs and mast cells in the skin of WT mice on P7, whereas it was not observed in the skin of WT mice with OIR on P17, confirming the release of mMCP6 (Figure 5B). mMCP6 levels were suppressed in cromolyn-treated WT mice compared with mice injected with PBS (Figure 5C). Nafamostat mesilate (NM), a specific inhibitor of tryptase (36), or mMCP6-neutralizing mAbs were injected i.p. into murine pups daily from P12 to P17 (Figure 5D). Administration of these tryptase inhibitors markedly reduced the number of neovascular nuclei in WT and BMCMC-treated *Kit^Wsh/Wsh^* pups on P17 (Figure 5E). To further confirm the role of mMCP6 in retinal neovascularization in *Kit^Wsh/Wsh^* mice, we injected recombinant mMCP6 on P12. Treatment of recombinant mMCP6 induced neovascularization in mast cell–deficient mice in a dose-dependent manner to a degree seen in WT pups (Figure 5F). When we transferred BMCMCs from WT mice into the OIR model of *Cpa3^Cre/+^* mice, retinal neovascularization was induced (Figure 5G). On the other hand, when BMCMCs generated from mMCP6-deficient mice were used, no retinal neovascularization was observed (Figure 5G), proving that mast cell tryptase (MCT) was essential for the pathogenesis of OIR in this murine model.

Since protease-activated receptor 2 (PAR2) is activated by tryptase (37), we examined the possible involvement of PAR2 in retinal neovascularization in the OIR model using PAR2-deficient mice. Unexpectedly, PAR2-deficient pups developed retinal neovascularization after exposure to relative hypoxia (Figure 5H).

### MCT induces expression of angiogenic factors in the retina.

**Figure 6:**
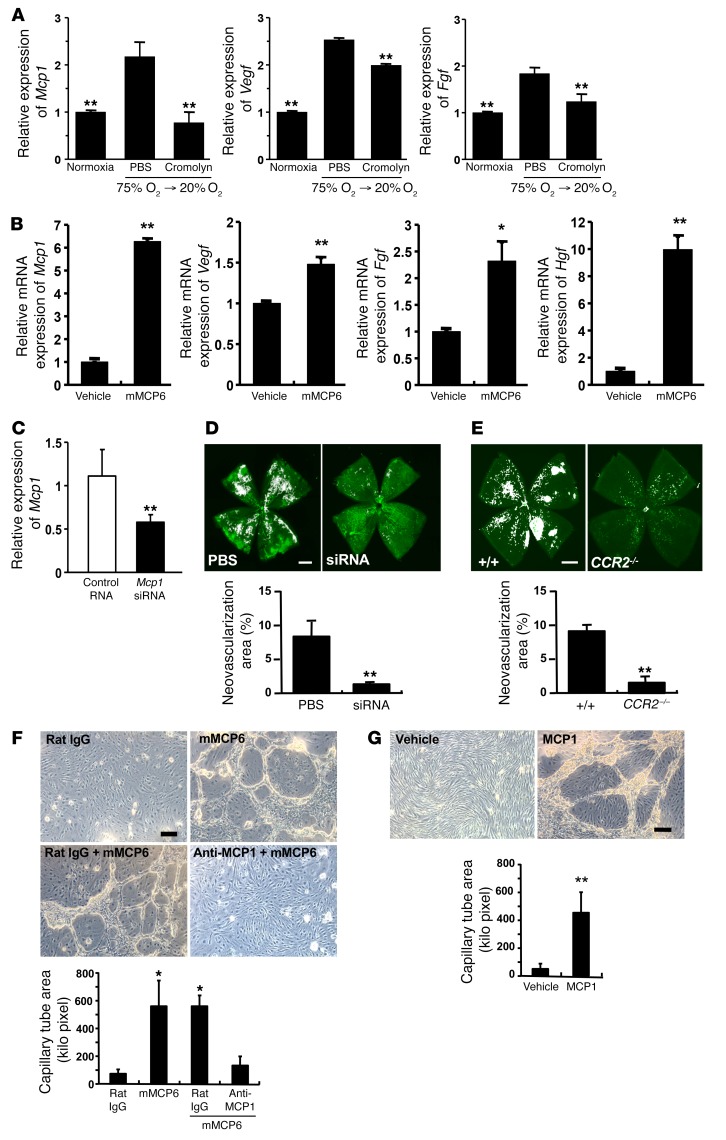
Tryptase directly induced the expression of angiogenic factors in retinal endothelial cells. (**A**) *Mcp1* mRNA expression was significantly increased in OIR mice. This was suppressed by administration of cromolyn, but administration of PBS alone did not have any effect. Other angiogenic factors, *Vegf* and *Fgf*, were upregulated in OIR mice and decreased by cromolyn treatment. On P11 and P12, WT mice were injected with PBS or cromolyn, and eyes were collected at 6 hours after the second administration on P12. *n* = 8 in each group. **P < 0.01 versus PBS-injected mice, Dunnett’s test. (**B**) Addition of recombinant mMCP6 into the culture of primary retinal endothelial cells induced *Mcp1*, *Vegf*, *Fgf*, and *Hgf* gene expression. *n* = 8 in each group. *P < 0.05; **P < 0.01 versus vehicle treatment, Mann-Whitney *U* test. (**C**) Schematic of *Mcp1* gene–silencing experiments. Intravitreal injection of *MCP1* siRNA effectively suppressed retinal *MCP1* expression. *n* = 8 in each group. **P < 0.01 versus control RNA-injected mice, Mann-Whitney *U* test. (**D**) Abnormal angiogenesis, following relative hypoxia, was suppressed by the specific inhibition of *MCP1* in the retina. Neovascularization area (%) was quantified in whole-mount specimens. *n* = 8 in each group. **P < 0.01 versus PBS-injected mice, Mann-Whitney *U* test. (**E**) Relative hypoxia induced the formation of new abnormal blood vessels (white areas) in WT mice (*n* = 14), but not in CCR2-deficent mice (*n* = 8). **P < 0.01 versus WT mice, Mann-Whitney *U* test. (**F**) Addition of recombinant mMCP6 into the culture of primary retinal endothelial cells induced typical tube formation. The effect of mMCP6 was suppressed by addition of anti-MCP1 mAbs. *n* = 4 in each group. *P < 0.05 versus rat isotype mAb, Dunnett’s test. (**G**) Addition of recombinant mouse MCP1 (10 ng/ml) into the culture of primary retinal endothelial cells induced typical vascular tube formation. *n* = 4 in each group. **P < 0.01 versus vehicle control, Mann-Whitney *U* test. Scale bars: 500 μm (**D**, **E**); 100 μm (**F**, **G**). All results are shown as mean ± SEM of values determined from 3 to 4 independent experiments.

To investigate the mechanism by which mast cells induce retinal neovascularization, we examined the expression of a number of angiogenic factors by real-time PCR. We found that monocyte chemotactic protein-1 (*Mcp1*) mRNA was highly expressed in the retina of the OIR model and that cromolyn completely blocked its expression (Figure 6A). *Vegf* and *Fgf* were also upregulated, but *Hif1a* and hepatocyte growth factor (*Hgf*) were not induced in the retina of OIR mice (data not shown). Cromolyn also decreased mRNA expression of *Vegf* and *Fgf* in the WT mice (Figure 6A). Recombinant mMCP6 added to primary culture of murine retinal microvascular endothelial cells enhanced mRNA expression of *Mcp1*, *Vegf*, *Fgf*, and *Hgf* (Figure 6B). Since MCP1 has been proposed as a key angiogenic factor of microvascular endothelium (38), we examined the role of MCP1 in retinal angiogenesis in the OIR model. Intravitreous injection of siRNA against *Mcp1* on P12 suppressed mRNA transcription of *Mcp1* (Figure 6C). The development of OIR was markedly abrogated by the injection of siRNA against *Mcp1* (Figure 6D). Since positive immunoreactions for CCR2 were observed at the tufts of the retina (Supplemental Figure 1; supplemental material available online with this article; https://doi.org/10.1172/JCI89893DS1), CCR2-deficient mice were used to further clarify the involvement of CCR2 in this OIR model. In CCR2-deficient mice, we also found that retinal neovascularization was markedly reduced when compared with that in WT mice (Figure 6E). To clarify the role of mMCP6 and MCP1 in retinal neovascularization, we checked whether mMCP6-induced tube formation of murine retinal microvascular endothelial cells was independent of retinal infiltration of monocytes and other leukocytes. Addition of recombinant mMCP6 produced clear capillary tube formation in growth factor–reduced Matrigel, and pretreatment with anti-MCP1 mAbs blocked the effect (Figure 6F). Recombinant MCP1 induced capillary tube formation comparable to that of mMCP6 (Figure 6G). Furthermore, as MCP1 is classically thought of as a chemotactic factor of monocytes/macrophages, infiltration of these cells was examined in the retina of WT mice. Few if any Ly6C-CD45 double-positive cells were observed within the retinal neovascular sprouts and tufts (Supplemental Figure 1). These results indicated that mMCP6 released from relative hypoxia-stimulated mast cells activated MCP1 production in retinal endothelial cells to induce abnormal angiogenesis in the retina, resulting in OIR.

### Mast cell distribution.

**Figure 7:**
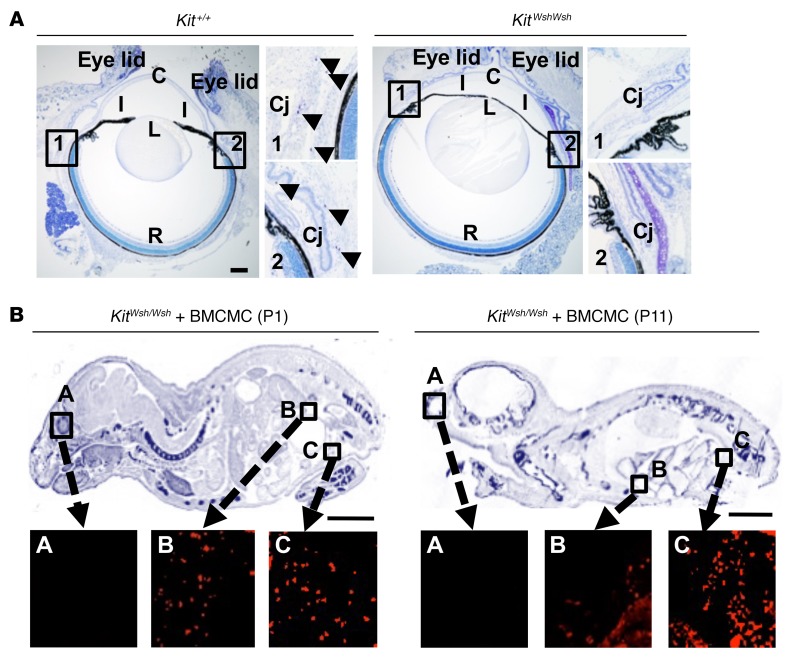
Mast cell distribution. (**A**) Toluidine blue staining of eyes and their surrounding tissue in naive mice. Mast cells were distributed in the conjunctiva (arrowheads) in WT mice. R, retina; Cj, conjunctiva; C, cornea; I, iris; L, lens; Cil, ciliary body. Scale bar: 500 μm. (**B**) Mast cell distribution in *Kit^Wsh/Wsh^* mice reconstituted with EGFP-BMCMC was investigated by immunohistochemistry. Whole-body sections were stained with 0.05% toluidine blue (upper panels) and with anti-GFP Abs (lower panels). Scale bars: 500 μm (**A**); 5 mm (**B**). Results shown are representative of 3 independent experiments (**A** and **B**).

We found mast cells in the connective tissue around the eyeball, but not in the retina of mice (Figure 7A). To investigate the mast cell distribution in BMCMC-injected *Kit^Wsh/Wsh^* recipients, we used BMCMCs derived from EGFP-transgenic mice. EGFP-positive cells were visualized by anti-GFP Abs and Alexa Fluor 594–conjugated anti-IgG Abs. EGFP-BMCMCs localized only to the peritoneal cavity on P11 (Figure 7B). On P17, 373 ± 97 BMCMCs were observed in the peritoneal cavity, and 42% of those had degranulated. These results indicate that mast cells were responsible for inducing the aberrant angiogenesis and retinal dysfunction in the OIR model. Furthermore, the data suggest that the angiogenic effect of mast cells did not require that they infiltrate into the retinal tissue, but indicate that they could be mediated by mMCP6 produced by mast cells residing in extraocular tissues. These results indicate that mMCP6 secreted from mast cells outside the eye must diffuse into the retina to induce neovascularization through upregulation of the angiogenic factors. It is well recognized that mast cell degranulation and release of chemical mediators, including tryptase, can cause a generalized increase in vascular permeability, fluid shifts out of the circulation, and subsequent anaphylactic shock. We therefore used i.v. injection of Evans blue to study vascular integrity in the OIR mouse model. Generalized leakage of dye out of the circulation and into the skin and brain was demonstrated in mice with OIR, but not in the controls (Supplemental Figure 2).

### Human preterm neonates with ROP have higher plasma MCT than gestational age–matched controls.

**Figure 8:**
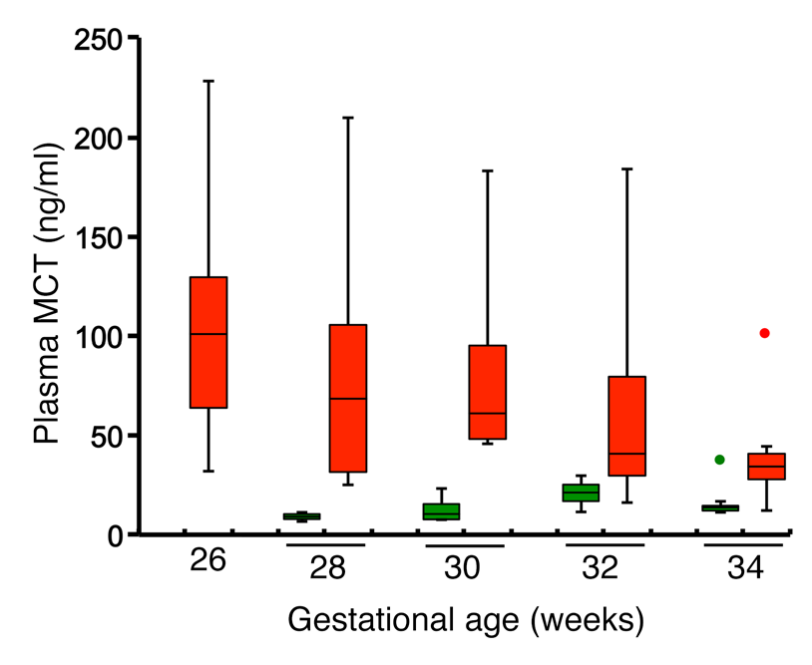
Plasma MCT levels of premature infants with and without ROP. Plasma levels of MCT were measured by ELISA. Obtained data are shown using a box plot that is composed of the median (solid line in each column), upper hinge, lower hinge, whiskers representing upper adjacent value or lower adjacent value, and far out values (green or red dot at 34 gestational weeks). Red boxes indicate MCT levels of preterm newborns with ROP (*n* = 8, average stage 3), and green boxes indicate MCT levels of those without ROP (*n* = 15). Results are from 3 independent measurements and compared by Mann-Whitney *U* test.

Finally, we measured plasma MCT levels of 23 preterm infants nursed in the neonatal intensive care unit until they achieved an oxygen saturation level of 90%. Examination of the fundi was performed weekly at the equivalent of 28 to 35 weeks gestation. Blood samples were collected from 8 preterm infants delivered at 22 to 24 weeks gestation who all developed ROP (average stage 3) and also suffered from bronchopulmonary dysplasia. The control group consisted of 15 preterm infants delivered at 28 to 34 weeks gestation, none of whom developed ROP or bronchopulmonary dysplasia. Blood samples for measurement of MCT were taken from the 2 groups at comparable gestational equivalent ages (28, 30, 32, and 34 weeks) (Figure 8). Median (interquartile range) plasma MCT of the preterm newborns with ROP at the equivalent of 28–32 weeks gestational age was 61.3 (32.8–95.7) ng/ml, 5-fold higher than that of control infants at equivalent gestational age who did not develop ROP (12.4 [8.8–21.9] ng/ml) (P = 0.001). Mild to moderate extraretinal fibrovascular proliferation was observed in most ROP patients. Partial retinal detachment was identified in one of the patients with ROP. These data suggest that in humans, as in mice, relative hypoxia induces mast cell degranulation and subsequent MCT release.

## Discussion

Our results prove for the first time, to our knowledge, that mast cells are indispensable for retinal neovascularization in both the mouse and rat models of OIR. Rodents lacking mast cells, either because of mutations in Kit (*Kit^Wsh/Wsh^*) or due to CPA3-driven Cre toxicity (*Cpa3^Cre/+^*), do not develop retinal neovascularization. However, upon reconstitution with mast cells, severe OIR can also be induced in these inherently mast cell–deficient animals. Second, we also show that activation of mast cells by relative hypoxia but not hyperoxia is via TRPA1, as the process can be blocked using the TRPA1 inhibitor HC-030031 (33). TRPA1 can therefore be considered an oxygen sensor that induces mast cell degranulation. TRPA1 is a primarily ion channel sensor of noxious stimuli and is sensitive to changes in oxygen tension (32). Although it has previously been shown that relative hypoxia after exposure to hyperoxia altered the expression of a diverse set of hypoxia-regulated genes (39), we clearly demonstrate that mast cells can trigger an “angiogenic switch” in OIR. It is interesting that it is not the initial change from low to high oxygen concentrations, but rather the subsequent relative hypoxia that triggers mast cell degranulation. Third, in these animal models of OIR, mMCP6 mediates spouting angiogenesis. The process can be inhibited by the mast cell stabilizer cromoyln and the specific tryptase inhibitor NM, and it can be induced in mast cell–deficient mice with recombinant mMCP6. The presence of mast cells close to the blood vessels and their involvement in angiogenesis at sites of cancer, inflammation, and tissue repair through the activation of endothelial cells have been well documented. Tryptase is known to degrade connective tissue matrix and collagen IV in vascular basement membranes (40). PAR2 has been proposed to mediate effects of MCT (41). However, in the present study, we could not demonstrate the necessity of PAR2 in OIR by using PAR2-deficient mice (42). It has been reported that pronounced intravitreal neovascularization develops in mice with conditional knockdown of PAR2 and that treatment with a PAR2 agonist accelerated normal revascularization (43). These findings suggest that PAR2 expressed in the retina functions as a modulator of oxygen-induced retinal inflammation. Therefore, MCT might not significantly affect the biological activity of PAR2 in the development of OIR. Further experiments using conditional PAR2 knockdown and treatment with PAR2 agonists may help to clarify whether this receptor has any modulatory role in mast cell–mediated retinal neovascularization in the murine model of OIR.

Weakening of the localized segments of vascular basement membrane by tryptase is consistent with the current concept of sprouting angiogenesis, which is initiated by pericyte detachment from the vessel wall and degradation of the solid support of the vascular basement membrane by enzymatic digestion. Pathological changes seen in OIR were associated with retinal dysfunction. We showed that mast cell–sufficient WT mice and mast cell–deficient mice injected with BMCMCs had lower or no amplitudes of b-waves and OP-waves in the ERG, suggesting functional damage to bipolar cells and amacrine/inner plexiform cells, but not to photoreceptor cells (44).

We also focused on the effect of MCP1 on retinal neovascularization in the OIR model. As previously reported, P17 retinal neovascularization induced in mice with embryonic disruption of the gene encoding *MCP1* was comparable to that in WT mice (45). As embryonic gene disruption might lead to unexpected compensation that appears as normal gene function, we used siRNA silencing of *MCP1* to more precisely examine MCP1 involvement in OIR. Intravitreous injection of siRNA suppressed both expression of *MCP1* mRNA and neovascularization. Moreover, OIR did not develop in MCP1 receptor CCR2-deficent mice. Tryptase induced capillary tube formation of primary retinal microvascular endothelial cells, as reported in human dermal microvascular endothelial cells (46), and pretreatment with anti-MCP1 Abs significantly suppressed tryptase-induced tube formation. Addition of MCP1 to primary retinal microvascular endothelial cells induced typical tube formation. Thus, we conclude that MCP1 was a key second messenger for the development of OIR and that it acted directly on the endothelial cells rather than via inducing the influx of monocytes.

In summary, our results show that MCT-mediated MCP1 production can promote proliferation of endothelial cells and vessel tube formation via an autocrine pathway, upregulating the expression of downstream factors responsible for angiogenesis, such as VEGF and FGF (47). Mast cell stabilizers blocked *Mcp1* and angiogenic growth factor expression and prevented the development of OIR, demonstrating that mast cells promote retinal neovascularization through a tryptase/MCP1 pathway.

Finally, we show that human neonates with ROP have significantly higher plasma MCT levels than gestational age–matched controls. MCT levels are used as a marker of mast cell activation in cases of anaphylaxis and mastocytosis. According to diagnostic criteria, serum MCT levels persistently exceed 20 ng/ml and can be as high as 200 ng/ml in patients with systemic mastocytosis (48). However, in healthy children and adults, normal MCT levels are less than 10 ng/ml (49). Plasma MCT levels in preterm neonates with ROP examined in the current study were as high as those of adult subjects with systemic mastocytosis. Human neonates with ROP also had bronchopulmonary dysplasia, suggesting that the increased number of MCT-positive mast cells in the lung contributes to high levels of MCT in preterm neonates with ROP (50). In addition to ROP, very preterm newborns are also at increased risk of lung damage and intracranial hemorrhage (51). We demonstrated that there is a generalized disturbance in vascular integrity in mice with OIR. A question requiring further research is whether the same disturbances of mast cell activity may also contribute to overt vascular diseases in the brain and other organs in very premature neonates. Our results suggest the therapeutic potential of mast cell stabilizers in the treatment of ROP. Mechanistic studies are difficult in human neonates for ethical reasons, but a simple proof-of-principle study would be a randomized blinded placebo-controlled trial to evaluate the effectiveness of mast cell stabilizers in preventing the development of retinal neovascularization in a group of at-risk preterm neonates. This would provide further evidence that the biological basis of ROP in humans is the same as in rodents.

Mast cells with specific granules brimming with neutral serine proteases are ideally placed as providing an interface between chronic inflammation and tissue remodeling. This study uses the eye as a window into understanding the process of postischemic reperfusion injury that occurs in OIR and ROP. The broader role of mast cells in other neonatal-related and reperfusion-related diseases might allow the development of novel therapeutic strategies for treating not only blindness, but also diseases as diverse as strokes and cancers (52, 53).

## Methods

### Animals.

C57BL/6-*Kit*^Wsh/Wsh^** mice were obtained from the RIKEN BioResource Center. C57BL/6J mice were purchased from Japan SLC. Mice with a *Kit*-independent defect in mast cells caused by the expression of Cre recombinase from the *Cpa3*, C57BL/6-*Cpa3^Cre/+^*, have been previously described (26). DA/Ham-*Kit^Ws/Ws^* rats and their WT littermates were purchased from Japan SLC. PAR2-deficient mice were supplied by Kowa Co. mMCP6-deficient C.129S-*Tpsb2^tm1.1Mfg^*/Mmnc mice were obtained from the Mutant Mouse Regional Resource Center (University of North Carolina, Chapel Hill, North Carolina, USA). EGFP-transgenic mice (C57BL/6 TgN(act-EGFP)OsbC14-Y01-FM131) were provided by M. Okabe (Genome Information Research Center, Osaka University, Osaka, Japan). TRPA1-deficient mice (B6;129P-*Trpa1^tm1Kykw^*/J) and CCR2-deficent mice (B6.129S4-*Ccr2^tm1Ifc^*/J) were supplied by The Jackson Laboratory.

### Animal model of OIR.

OIR was induced as previously described (24). On P7, animals were exposed to 75% O_2_ with their nursing dam in a sealed chamber. Pups remained in the chamber for 5 days (P7–P12) and were then placed in an ambient atmosphere for an additional 5 days (P12–P17).

### Mast cell injection.

As described previously (54), BM cells isolated from femora and tibiae were cultured in α-MEM with 10^–4^ M 2-ME (Sigma-Aldrich), 10% FCS, and 10% pokeweed mitogen-stimulated spleen conditioned medium for 4 to 8 weeks. BMCMCs (10^6^ cells/20 μl) obtained from WT mice, EGFP-transgenic mice, or mMCP6-deficient mice were injected i.p. in pups on P1 or P2. For rat experiments, peritoneal mast cells (10^6^ cells/20 μl) obtained from WT rats were injected following the same protocol.

### Evaluation of retinal neovascularization.

For whole-mount analysis, eyes were enucleated and fixed for 1 hour in 4% paraformaldehyde. Retinas were dissected and stained overnight in Alexa Fluor 488–conjugated *Griffonia simplicifolia* isolectin B4 (Molecular Probes). Retinal flat mounts were generated, and images were obtained using a BIOREVO system (Keyence). Total area, the number of clock hours of neovascularization, and avascular area were quantified with Photoshop (Adobe Systems).

### Histology and immunohistochemistry.

Eyes were enucleated from mice on P17 and fixed in Davidson’s fixative overnight. Serial 6-μm paraffin-embedded axial sections were stained with H&E. The nuclei on the vitreous side of the internal limiting membrane were counted. To examine mast cell distribution in mice and rats, toluidine blue or chloroacetate esterase staining was performed on paraffin sections of the dorsal skin (4 μm) and the retina (6 μm). Mast cells between epithelium and panniculus carnosus were counted under a microscope. For immunohistochemistry, eye sections were incubated overnight at 4°C with anti–PECAM-1 Abs (Santa Cruz Biotechnology Inc., catalog sc-1506). To visualize the target cells, sections were treated with the second Ab conjugated with biotin (Jackson ImmunoResearch; catalog 705-065-147). Serial 5-μm frozen skin sections were incubated overnight at 4°C with biotin-conjugated anti-mMCP6 Abs (Bioss, catalog bs-2726R-Biotin). To specifically visualize BMCMCs derived from EGFP-transgenic mice in whole-body sections of *Kit^Wsh/Wsh^* mice, samples were frozen with SCEM (Leica Microsystems). Sections (5 μm) were incubated overnight at 4°C with anti-GFP Abs (Invitrogen, catalog G10362), followed by Alexa Fluor 594–conjugated goat anti-rabbit IgG (Invitrogen, catalog A31631). Mast cells in the peritoneal cavity per section were counted.

### ERG.

As described previously (55), animals were dark adapted overnight and then anesthetized with ketamine hydrochloride (80 mg/kg) (Daiichi Sankyo Propharma) and xylazine hydrochloride (16 mg/kg) (Bayer Medical). After corneal treatment with 0.4% tropicamide (Nitten) and 0.4% oxybuprocaine hydrochloride (Santen Pharmaceutical), a contact lens electrode was placed on the eye, and reference and ground electrodes were placed in the mouth and on the tail, respectively. ERG was recorded with a PuREC system (Mayo Corporation) on P19.

### Degranulation assay.

BMCMCs derived from WT mice or TRPA1-deficent mice were cultured with or without 10 ng/ml cromolyn or various doses of HC-030031 for 5 days in 75% oxygen and then in 20% oxygen for 12 hours. Mast cell degranulation was measured by release of β-hexosaminidase using p–Nitrophenyl-*N*-acetyl-β-d-glucosaminide dissolved in 0.1 M sodium citrate (Sigma-Aldrich) as a substrate (56). After mixing culture supernatants and cell lysates with the substrate, the reaction was terminated by addition of a 200 mM glycine solution. Absorbance was measured at 414 nm.

### Drug administration.

We injected 20 μl of a mast cell stabilizer, cromolyn (57) (50 mg/kg, Sigma-Aldrich), a specific inhibitor of tryptase NM (36) (1 mg/kg, Torii Pharmaceutical Co.), or anti-mMCP6 mAbs (15 μg/kg, R&D Systems, catalog MAB4288, clone 286828) i.p. into pups once a day. Mice were administrated i.p. with various doses (20 μl) of recombinant mMCP6 (R&D Systems) immediately after the shift from hyperoxia to normoxia on P12. For TRPA1 inhibition, HC-030031 (300 mg/kg, Sigma-Aldrich) was i.p. injected into pups once a day, from P11 to P16. siRNA injection was conducted according to the method described previously (58). Briefly, control or *MCP1*-specific siRNA (Santa Cruz Biotechnology Inc., catalog sc-37007 and catalog sc-43914, respectively) was mixed with TransIT-TKO (Mirus Bio) and water, then 100 pmol siRNA in 1 μl was injected intravitreally on P12.

### Real-time PCR analysis.

Frozen retinas were homogenized with a multi-beads shocker (Yasui Kikai Corp.), and total RNA was extracted. mRNA expression of angiogenic factors was analyzed by real-time PCR using target-specific primers (Takara Bio).

Primary retinal microvascular endothelial cells isolated from C57BL/6 mice (Cell Biologics Inc.) were cultured in a specified endothelial cell medium supplement kit (Cell Biologics Inc.) and passaged no more than 3 times. After washing with PBS, recombinant mMCP6 was added to the culture (final concentration of 300 ng/ml) and incubated overnight. Cells were collected and processed for real-time PCR analysis. To assess effects of MCP1, 10 μg/ml rat anti-MCP1 mAbs (R&D Systems, catalog MAB479, clone 123616) were added into the culture. Rat IgG (Abcam, catalog ab18450, clone RTK2758) was used as an isotype control.

### ELISA.

Serum mMCP6 levels were measured using an ELISA kit (USCN Life Science, catalog E91070Mu). Plasma tryptase levels in infants were measured with a Human Tryptase Beta 2 ELISA Kit (USCN Life Science, catalog SED781Hu).

### TRPA1 analysis.

Flow cytometric analysis of TRPA1 was performed with rabbit anti-TRPA1 Abs (Novus Biologicals, catalog NB110-40763) and fluorescein-conjugated second Abs using BMCMCs derived from C57BL/6 mice that were treated with 0.1% Triton X-100 for permeabilization of cell membrane. Rabbit serum was applied instead of first Abs as controls. For immunoblotting, whole cell lysates were isolated from BMCMCs using a RIPA buffer. Immunoblotting was performed with rabbit anti-TRPA1 Abs and HRP-linked anti-rabbit IgG (Cell Signaling Technology, catalog 7074S). To detect loading controls, rabbit anti-GAPDH Abs (Cell Signaling Technology, catalog 2118S) were used after stripping at 50°C for 20 minutes. SDS-PAGE was performed using 7.5% gels, and positive reactions were visualized using Immobilon Western Chemiluminescent HRP Substrate (Millipore). As a positive control, we used protein samples isolated from the mouse brain membrane.

### Endothelial cell tube formation.

Primary retinal microvascular endothelial cells were cultured in growth factor–reduced Matrigel (Corning) as described in a previous report (46). Briefly, cells were resuspended at a concentration of 2 × 10^4^ cells/500 μl in 24-well plates and incubated at 37°C in a humidified atmosphere of 5% CO_2_ and 95% air. Three days after the initiation of culture, 10 μg/ml anti-MCP1 mAbs (R&D Systems) or isotype mAbs (Abcam) were added and 300 ng/ml mMCP6 was added on days 4 and 5. At 16 hours after the second addition of mMCP6, cells were fixed with 4% paraformaldehyde and the area of capillary tubes was quantitated with ImageJ ver. 1.51 (NIH). In the other experiment, 10 ng/ml recombinant mouse MCP1 (R&D Systems, catalog 479-JE) was added on days 4 and 5. At 7 days after the second addition of MCP1, cells were fixed.

### Patient study.

Plasma samples were stored at −20°C until use. MCT levels were measured by using an ELISA kit (Uscn Life Science, catalog SED781Hu) according to the manufacturer’s instructions.

### Statistics.

All data were compared by 1-way ANOVA with Tukey’s test, Dunnett’s test, or Mann-Whitney *U* test as described in each figure legend. *P* values of less than 0.05 were considered significant. All animal data are presented as mean ± SEM of at least 3 independent experiments. Human MCT levels are quoted as median and interquartile ranges and differences between groups determined by Mann-Whitney *U* test.

### Study approval.

All animal experiments were conducted in accordance with the guidelines of and approved by the University Animal Care and Use Committee of the Tokyo University of Agriculture and Technology (project approval: no. 24-93 and no. 28-84). Blood samples were collected from preterm infants being cared for in the Neonatology Unit with the approval of the ethical committee of Tokyo Metropolitan Children’s Medical Center and with the informed consent of the babies’ parents (project approval no. H25-32).

## Author contributions

KM, N. Okamoto, KK, SI, SY, AM, KJ, KO, YA, and HJ performed the experiments, evaluated data, and applied statistical analysis. YM contributed to experiments with TRPA1-deficient mice. N. Onai and TO contributed to experiments with CCR2-deficient mice. MK, RK, KY, and UK took care of premature infants and collected blood samples. EN performed ophthalmologic examinations on premature newborns. KM and N. Okamoto wrote the draft. PDA and AT collected data and extensively reviewed and revised the paper. HM conceived and directed the project. All the authors had the opportunity to discuss the results and comment on the manuscript.

## Supplementary Material

Supplemental data

## Figures and Tables

**Table 2:**
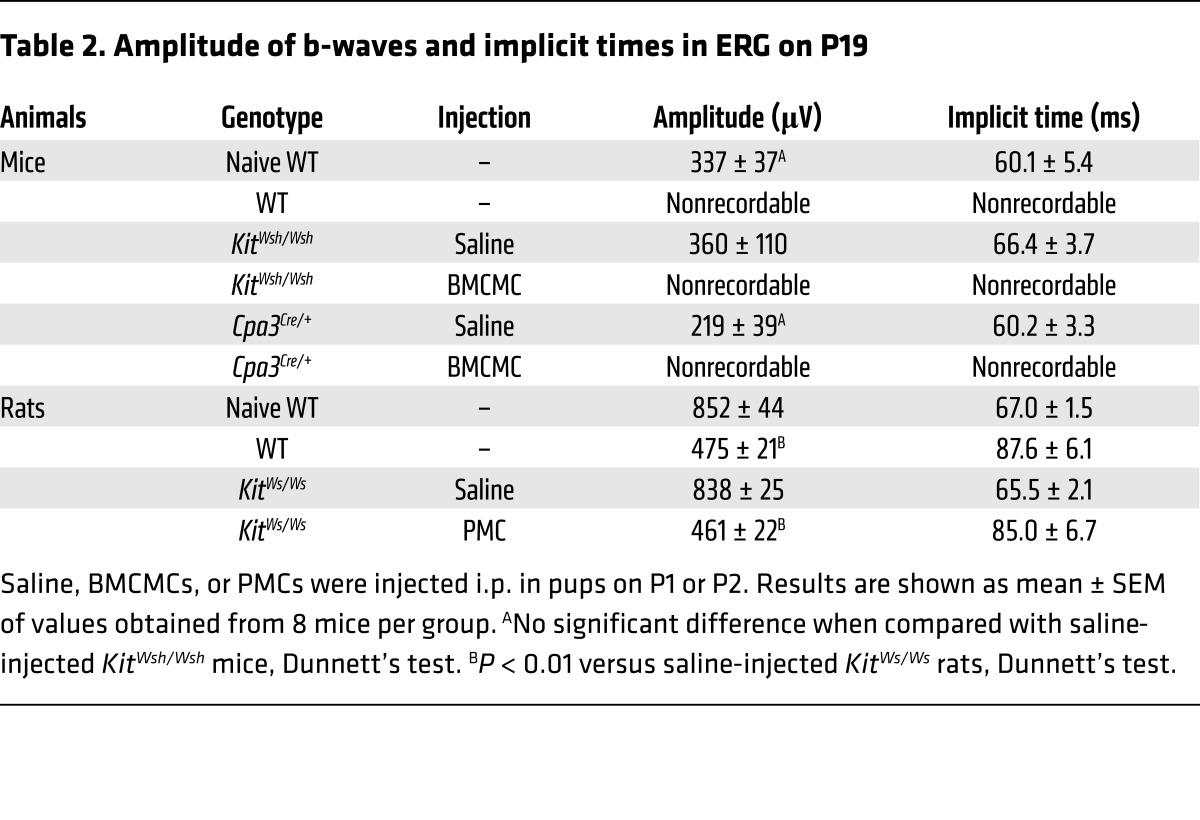
Amplitude of b-waves and implicit times in ERG on P19

**Table 1:**
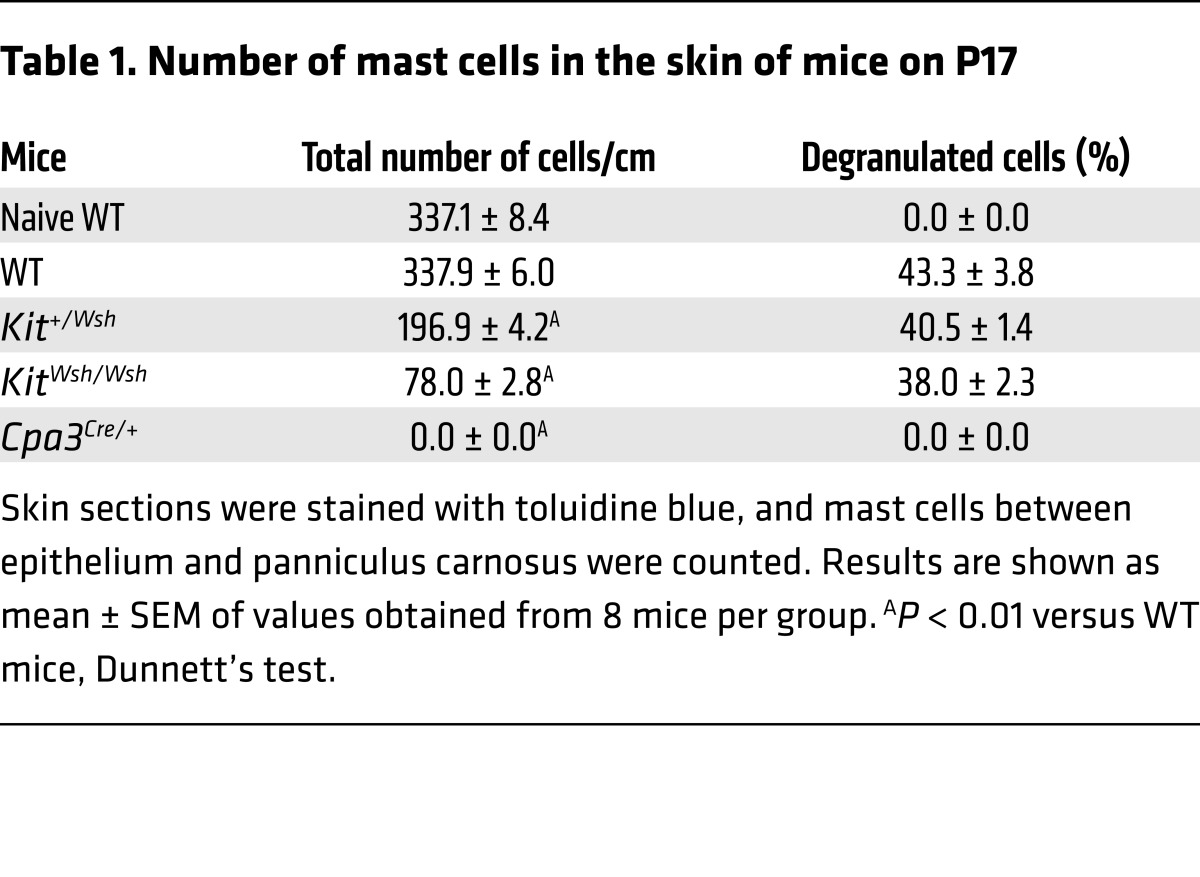
Number of mast cells in the skin of mice on P17

## Version 1 10/09/2017

Electronic publication

## Version 2 11/01/2017

Print issue publication
