# Supplementary material for: Mast cell hyperactivity underpins the development of oxygen-induced retinopathy
Source: J Clin Invest. 2017 Oct 9;127(11):3987–4000. doi: 10.1172/JCI89893 (PMC5663365; doi:10.1172/JCI89893)
Supplement: Supplemental data [file jci-127-89893-s001.pdf]

Supplemental Figures and Figure legends

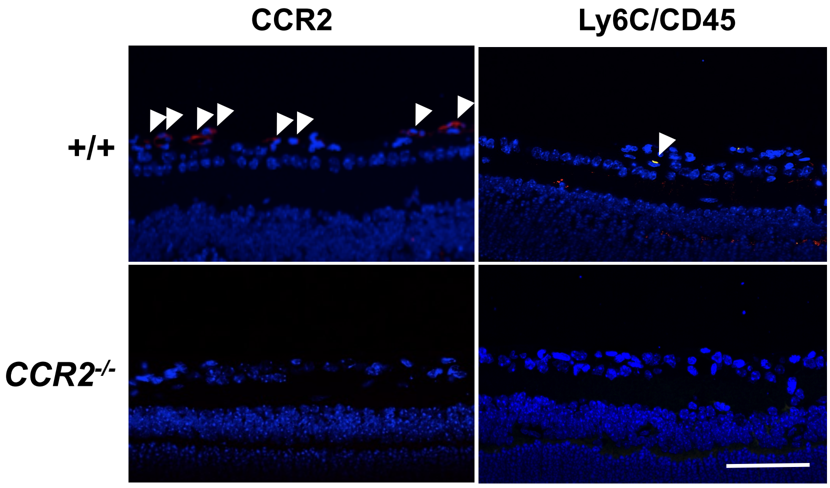

Supplemental Figure 1. Lack of infiltration of monocytes and CCR2 expression in the retina of wild type mice and CCR2-deficent mice on P17. Multiple staining methods were applied using Ly6C (red), CD45 (green), CCR2 (red), and DAPI (blue). Paraffin-embedded sections (5 μm) of the retina were stained with rabbit anti-mouse Ly6C Ab (Abcam, #ab-15627) and rat anti-mouse CD45 mAb (BioLegend, #103101), followed by Alexa Fluor 647-conjugated anti-rabbit IgG Ab (Life Technology, #A21245) and Alexa Fluor 488-conjugated anti-rat IgG Ab (Life Technology, #A11006). The other sections were stained with rabbit anti-mouse CCR2 Ab (Santa Cruz, #sc-30032), followed by Alexa Fluor 647-conjugated anti-rabbit IgG Ab. Prolong Gold antifade reagent with DAPI (Invitrogen, #P36835) was used for a nuclear counterstain. Results show representative of three independent experiments. Ly6C-CD45 double positive cells were colored yellow (arrowheads). Scale bar, 100 μm.

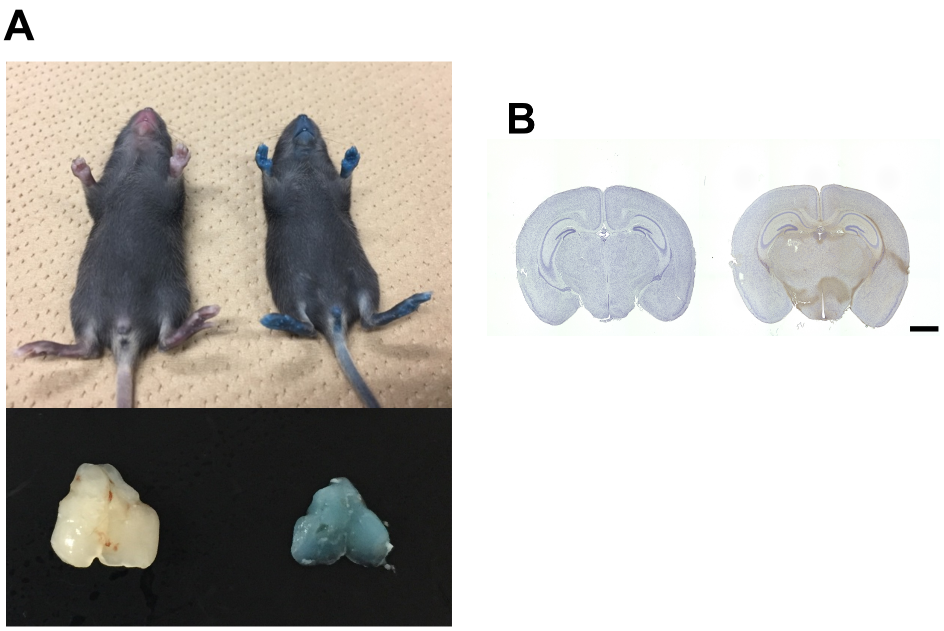

Supplemental Figure 2. Generalized increase in vascular permeability of mice with OIR. (**A**) C57BL/6 mice were injected i.p. with 30 μl of 0.5% Evans blue (Sigma-Aldrich, #E-2129) on P12 and sacrificed on P17. Extravascular infiltration of dye was apparent in the skin and lung of mice with OIR (right), but not in age-matched controls (left). (**B**) To evaluate blood-brain barrier, IgG staining of the brain was carried out. Paraffin-embedded sections (5 μm) of the brain were stained with biotinated goat anti-mouse IgG Ab (Santa Cruz, #sc-2039), and incubated with peroxidase-conjugated streptavidin (Dako, #P0397). After lightly washing, the sections were counterstained with Meyer's hematoxylin. Clear staining was observed in the brain of mice with OIR (right) as compared with that of controls (left). *n* = 6 in each group. Results show representative of two independent experiments. Scale bar, 1 mm.
